# Antidiabetic Drug Metformin Ameliorates Depressive-Like Behavior in Mice with Chronic Restraint Stress via Activation of AMP-Activated Protein Kinase

**DOI:** 10.14336/AD.2019.0403

**Published:** 2020-02-01

**Authors:** Heng Ai, Weiqing Fang, Hanyi Hu, Xupang Hu, Wen Lu

**Affiliations:** ^1^Department of Physiology, Hangzhou Medical College, Hangzhou, Zhejiang, China; ^2^Department of Pharmacy, Women's Hospital, School of Medicine, Zhejiang University, Zhejiang, China; ^3^Department of Ophthalmology, Sir Run Run Shaw Hospital, Zhejiang University, Hangzhou, China; ^4^Department of Neurobiology, Key Laboratory of Medical Neurobiology of Ministry of Health of China, Zhejiang University School of Medicine, Zhejiang, China; ^5^Department of Biochemistry and Molecular Biology, Hainan Medical University, Haikou, Hainan, China

**Keywords:** depression, metformin, chronic restraint stress, AMP-activated protein kinase, Compound C, antidepressant

## Abstract

Depression is one of the most prevalent neuropsychiatric disorders in modern society. However, traditional drugs, such as monoaminergic agents, have defect showing lag response requiring several weeks to months. Additionally, these drugs have limited efficacy and high resistance rates in patients with depression. Thus, there is an urgent need to develop novel drugs or approaches for the treatment of depression. Here, using biochemical, pharmacological, genetic and behavioral methods, we demonstrate that metformin imparts a fast-acting antidepressant-like effect in naïve mice as well as stressed mice subjected to chronic restraint stress model. Moreover, inhibition of AMP-activated protein kinase (AMPK) activity by compound C or knock down of hippocampal AMPKα occluded the antidepressant-like effect induced by metformin. Our results suggest that metformin may be a viable therapeutic drug for the treatment of stress-induced depression via activation of AMPK.

Chronic stress causes pathophysiological and psychological alterations associated with mental illness, leading to neuropsychiatric disorders including depression [1-3]. Based on estimation and reports, depression is one of the most prevalent neuropsychiatric disorders, leading to serious disability and affecting more than 350 million people worldwide [4]. Currently, depression is treated with monoaminergic agents. However, a major limitation of the present pharmacotherapies exist, showing these drugs may take several weeks or even months to elicit a therapeutic response [5, 6]. Preclinical and clinical findings demonstrate that glutamatergic agents, e.g., ketamine, which blocks N-methyl-D-aspartate receptors, produce rapid and long-lasting antidepressant effects [7-10]. However, cognitive impairment and psychotomimetic symptoms associated with the application of ketamine hinder its clinical use [11], thereby strengthening the demanding need to develop alternative fast-acting antidepressants for depression.

Metformin is the first-line drug for glycemic control, as it effectively reduces hepatic glucose production and improves insulin sensitivity in Type 2 diabetes mellitus in the clinic for more than five decades without stimulating insulin secretion, additional weight gain, or causing hypoglycemia [12-14]. Importantly, accumulating evidence has shown that metformin imparts numerous health benefits beyond its original therapeutic use in several neurological diseases, including Alzheimer’s disease, autism spectrum disorders and cerebral ischemia [15-19]. A previous study has shown that metformin may alleviate depression in diabetic patients, although the detailed mechanism remains to be explored [20]. Notably, chronic treatment with metformin promotes hippocampal neurogenesis [21]; this process is impaired during stress-induced depression, and elevation of neurogenesis counteracts with depression [22, 23]. However, the effect of acute metformin treatment on depression in experimental models remained poorly understood. Given that metformin is a Food and Drug Administration-approved drug in clinical use for several decades, it would be of great interest to explore the effects of metformin on depressive-like behavior.

AMP-activated protein kinase (AMPK), an evolutionarily conserved serine/threonine heterotrimeric kinase, is activated by phosphorylation of threonine 172 (T172) in the catalytic α-subunit in response to various stressors, such as hypoxia, glucose deprivation, and oxidative stress [24, 25]. AMPK is abundantly expressed in cultured hippocampal neurons as well as in the hippocampus *in vivo* [26, 27]. AMPK is the main energy sensor *in vivo*, and acts as a metabolic master switch, which is required for maintenance of energy homeostasis [25]. Interestingly, energy metabolism dysfunction is one of the most consistent features observed in psychiatric disorders [28]. Notably, activation of AMPK ameliorates stress-induced memory deficits [29]. In addition, metformin is a well-recognized AMPK activator both *in vitro* and *in vivo* [30]. Considering that metformin has numerous beneficial properties, we herein aimed to determine whether metformin could alleviate depressive-like behavior in mice submitted to chronic stress via activation of AMPK.

In the present study, we found that acute treatment of metformin indeed mitigates depressive-like behavior in mice. In addition, this beneficial effect of metformin on depressive-like behavior endured for at least 5 days but was diminished at 7 days post treatment. Moreover, systematic application of compound C, which is an inhibitor for AMPK, eliminated the ability of metformin in reducing depressive-like behavior. In line with the results from compound C, knock down of AMPKα in hippocampus by adeno-associated virus occluded the impact of metformin on depressive-like behavior. This work suggests that metformin may be a potential therapeutic drug for the treatment of depression and thus it is worth to be further verified in preclinical and clinical experiments in the future.

## MATERIALS AND METHODS

### Animals

All behavioral or biochemical experiments were conducted with 10-to 12-week-old male C57BL/6J mice purchased from Shanghai SLAC laboratory Animal Co. Ltd and raised in the laboratory for at least one week of adaption. All experiments were performed and analyzed blind to treatment. Four mice were housed per cage in a temperature and humidity-controlled environment (23±2°C, lights on 07:00-19:00) with ad libitum access to food and water. The protocols for the experiments with mice were approved by the Institutional Animal Care and Use Committee at Hainan Medical University and Hangzhou Medical College and the guidelines conform to the National Institutes of Health Guide for the Care and Use of Laboratory Animals. Efforts were made to minimize animal suffering during the experiments. All surgery and sacrifice were performed under sodium pentobarbital anesthesia.

### Stereotaxic injection of adeno-associated viruses (AAVs)

Six-week old male C57BL/6J mice were anesthetized with 1% pentobarbital (Sigma-Aldrich, St Louis, MO, USA) by intraperitoneal injection. The following stereotaxic coordinates for the hippocampus were used: 1.8 mm anterior from bregam, 1.6 mm lateral from midline, and 1.6 mm vertical from the cortical surface. One microliter adeno-associated virus (AAV) 2/8 serotype (1.31 × 10^13^ virus particles/mL) was injected into each side at a speed of 0.2 μl per minute. The pipette was held in the injection site for additional 6 min after injection and then gradually withdrawn. The animals were maintained on a heating pad throughout the surgery and were returned to their home cages after recovery. The nonspecific sequence used as a control is 5-TTCTCCG AACGTGTCACGT-3 and the sequence targeting mouse AMPKα was 5-GAGGAGAGCTATTTGATTA-3, which were inserted into the pAKD-CMV-betaGlobin-eGFP-H1-shRNA vector. AAVs were packaged by Obio Technology, Shanghai, China.

### Drugs and antibodies

Metformin hydrochloride (D150959) was purchased from Sigma-Aldrich (St Louis, MO, USA) and dissolved in sterile saline. Compound C (6-[4-(2-Piperidin-1-yl-ethoxy)-phenyl)]-3-pyridin-4-yl-pyyrazolo[1,5-a] pyrimidine) was obtained from Tocris (Cat. No. 3093) and dissolved in dimethyl sulfoxide and further diluted with sterile saline. Metformin and compound C (10 mg/kg) were injected intraperitoneally. Phospho-Acetyl-CoA Carboxylase (Ser79) (3661, 1:500), Acetyl-CoA Carboxylase (C83B10) rabbit mAb (3676, 1:1000), AMPKα (D63G4) rabbit mAb (5832, 1:1000) and Phospho-AMPKα (Thr172) (40H9) rabbit mAb (2535, 1:1000) were obtained from Cell signaling technology (Beverly, MA, USA). The mouse monoclonal antibody β-actin (A1978, 1:5000) was the product of Sigma-Aldrich (St Louis, MO, USA). HRP-conjugated secondary antibodies for western blot were diluted as 1: 10,000, and were from Thermo Fisher Scientific (Waltham, MA, USA).

### Chronic restraint stress (CRS)

Chronic restraint stress was adapted from our previous acute restraint stress protocol [31]. Mice were subjected to CRS by placement in 50-mL conical tubes with holes for air flow for 3 h (09:00-12:00) per day for 14 consecutive days. The animals were not physically compressed and did not experience pain.

### Measurement of corticosterone level

Blood samples were harvested immediately after CRS. The sera were collected by centrifugation at 3000 × g for 10 min at 4°C. The serum corticosterone level was measured by an ELISA kit according to the manufacturer’s instructions (CSB-E07969m, CUSABIO, Wuhan, China).

### Open field test (OFT)

Open field was performed as described previously [31]. Mice were continuously monitored using Smart Software (Smart 3.0, Panlab) after being placed in a white plastic arena (45 × 45 × 45 cm) in a room with dim light for 10 min. The total distance, and number of entries into the center of the arena (20 × 20 cm) were recorded.

### Forced swimming test (FST)

Mice were placed individually in a 4L glass chamber filled with 25 cm of water (temperature 24-26°C) for 6 min. The lengths of periods of immobility or struggling during the 6-min test period were measured as previously described [31].

### Tail suspension test (TST)

The protocol was carried out as described previously with minor modification [32]. Mice were suspended by the tail from a metal rod using adhesive tape. The rod was fixed 45 cm above the surface. Each mouse was positioned at least 15 cm away from the nearest object. The test sessions lasted 6 min, and the immobility time was determined by a skilled observer. The mice were regarded as immobile only when they hung passively and fully motionless.

### Sucrose preference test (SPT)

SPT was conducted as reported previously [33]. Mice were single housed and habituated with two bottles of water for 2 days, followed by two bottles of 2% sucrose for 2 days. The mice were then water deprived for 24 h and then exposed to one bottle of 2% sucrose and one bottle of water for 2 h in the dark phase. The sucrose consumption ratio was calculated by dividing the total consumption of sucrose by the total consumption of both water and sucrose.

### Preparation of lysate from hippocampal tissue

The procedure was performed as described in previously [34]. Briefly, the hippocampal tissues from mice were rapidly dissected and homogenized in ice-cold RIPA buffer containing 10 mM Tris, pH 7.4, 150 mM NaCl, 1 mM EDTA, 0.1% SDS, 1% Triton X-100, and 1% SDS (Beyotime) with Protease Inhibitor and Phosphatase Inhibitor and centrifuged at 16,000 × g for 20 min. Proteins in the supernatant were quantified using a BCA Protein Assay Kit from Thermo Fisher Scientific (Waltham, MA, USA), denatured by boiling in SDS sample buffer for 5 min.

### Western blot

Western blot was performed according to our previous protocol [31, 35]. Briefly, equal amounts of protein (20μg) were loaded onto 10% SDS-PAGE and then transferred to nitrocellulose membranes (Protran BA 85, Whatman, GE). The membranes were incubated in blocking buffer (2.5% BSA in TBST) for 1 h at room temperature, and then incubated with various primary antibodies in blocking buffer overnight at 4°C. After extensive wash with TBST for four times, the blots were incubated with HRP-conjugated secondary antibody for 1 h in blocking buffer at room temperature. Following extensive washes for four times with TBST, the blots were detected by chemiluminescence with ECL reagent (Bio-Rad). Densitometry analysis was performed with Quantity One software (Bio-Rad).

### Statistical analysis

Sample sizes were estimated on the basis of our past experience in performing similar experiments or previous reports by others. Mice were randomly assigned to treatment groups. Analyses were performed in a manner blinded to the treatment in all behavioral experiments. Statistical analysis was performed using GraphPad Prism 6 (GraphPad Software, La Jolla, CA, USA). All statistical graphs represent the mean ± standard error of the mean (SEM). Normal distributions of data were examined by the Kolmogorov-Smirnov test. Similarity in variance between groups was tested by Fisher’s (two groups) or Bartlett’s (multiple groups) tests. Statistical analysis was performed either with a two-tailed unpaired Student t-test when only two groups were compared, a one-way or two-way ANOVA analysis followed with Bonferroni post hoc test when indicated. Significance was assigned at P ≤ 0.05.

**Figure 1.**
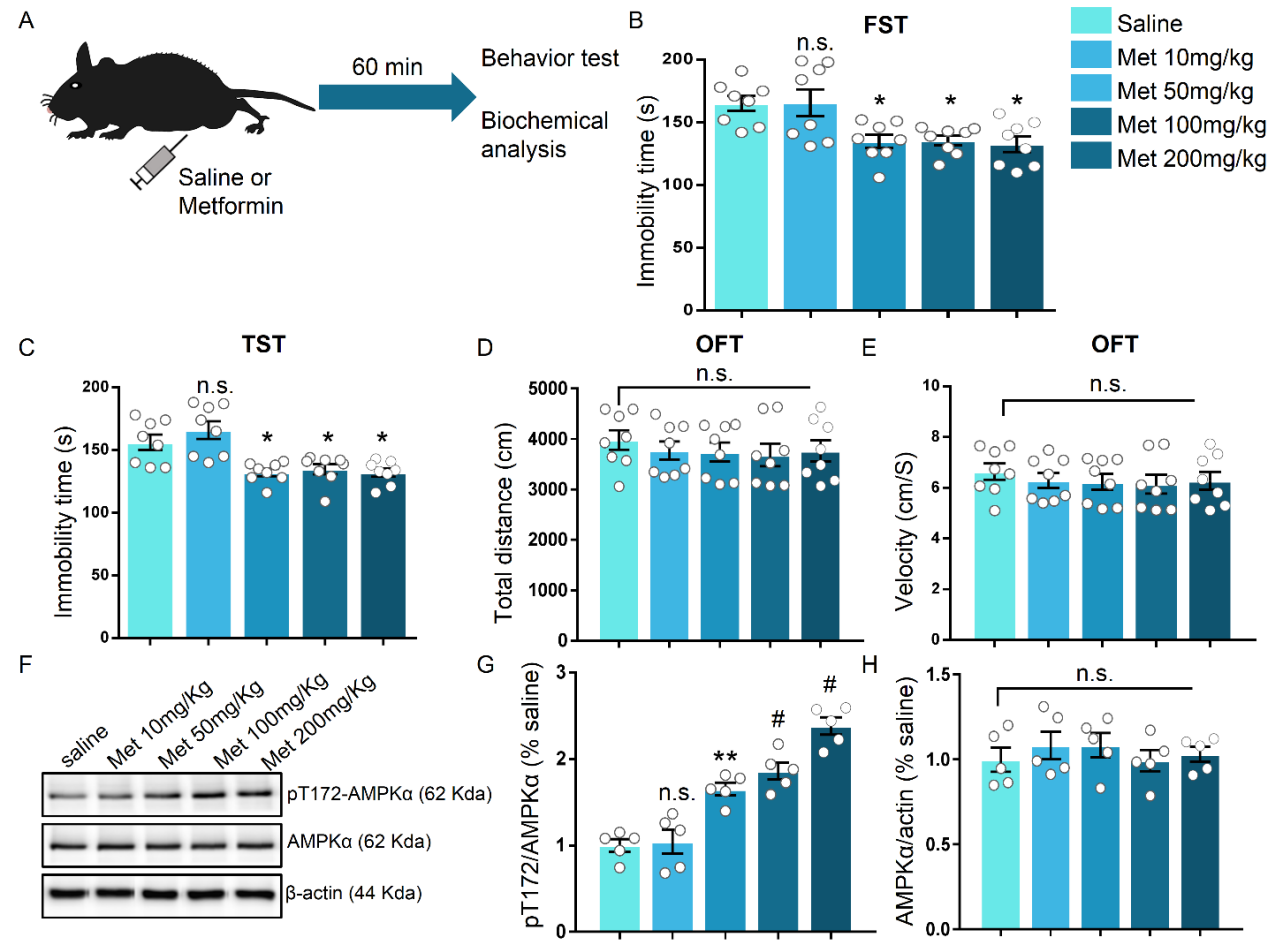
**Dose-dependent antidepressant-like effect of metformin in mice**. **(A)** Schematic illustration of the experimental set up. **(B-C)** Immobility time of mice after acute treatment of various doses of metformin (intraperitoneal injection) in FST (B) and TST (C), respectively. Data are shown as the mean immobility time ± SEM. Independent groups of mice were used for each behavioral test (n = 8 mice per group). **(D-E)** Mice receiving different doses of metformin were subjected to the open field test (OFT). The mean distance travelled (D) and the velocity (E) were recorded (n = 8 mice per group). **(F)** Immunoblots of hippocampal lysate from mice treated with various doses of metformin without behavioral test. The relative levels of p-AMPK (pT172) and AMPKα were analyzed, β-actin served as a loading control. **(G-H)** Quantification of fold changes in pT172 (G) and AMPKα (H) levels in the hippocampus, n = 5 mice per group. One-way analysis of variance (ANOVA) with Bonferroni post-hoc analysis. *P < 0.05, **P < 0.01, ^#^P < 0.001, n.s. represents not significant. Data are presented as mean ± SEM.

## RESULTS

### Metformin exerts antidepressant effects on mice in a dose- and time-dependent manner

**Figure 2.**
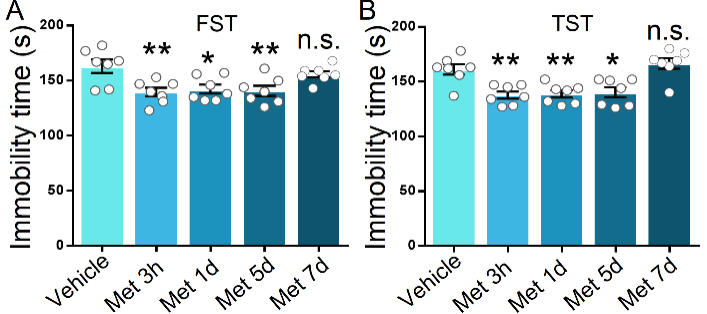
**Time course of metformin-mediated antidepressant-like behavioral effects**. After treatment with metformin (50 mg/kg, intraperitoneal injection), the immobility time of C57BL/6 mice in FST (A) and TST (B) at different time points was analyzed, respectively. Independent groups of mice were used for each behavioral test and each time point to avoid behavioral habituation (n = 7 mice per group). One-way analysis of variance (ANOVA) with Bonferroni post-hoc analysis. *P < 0.05, **P < 0.01, n.s. represents not significant. Data are presented as mean ± SEM.

In the first step, we evaluated the effects of metformin on mice by depressive-related behavior. Metformin was systematically injected into mice 1 h before behavior test (Fig. 1A). Following single injections of various doses of metformin, the anti-depressant effect was indicated by the responses in the forced swimming test (FST) and tail suspension test (TST), two widely used behavioral tests of depression. The FST focuses immobility time after mice being placed in a tank filled with water from which animals cannot escape, while the TST has a common theoretical basis and behavioral measure with the FST but avoids motor dysfunction that may interfere with evaluation in a FST [36, 37]. In the group that received metformin > 10 mg/kg, the immobility time in the FST and TST was significantly decreased compared to that in the vehicle-injected or 10 mg/kg metformin-injected groups (F (4, 35) = 6.226, P < 0.001 for FST; F (4, 35) = 9.961, P < 0.001 for TST; Fig. 1B, C). However, no effect was observed on locomotor activity as measured by the open field test in the mice with vehicle or metformin injections (Fig. 1D, E), suggesting that metformin has a minimal impact on the motor function of mice when administered in the range of 10 mg/kg to 200 mg/kg. Previous studies demonstrate that metformin can penetrate the brain and activate AMPK [38, 39]. To verify the effect of metformin on the brain in our model, we harvested the hippocampus of another batch of mice that received vehicle or metformin injection but without behavior test. By assessing the phosphorylation level of T172 in AMPK (pT172 level), we found that various doses of metformin (with the exception of 10 mg/kg) induced a robust increase in the pT172 level as compared to that induced by the vehicle (F (4, 20) = 33.94, P < 0.001; Fig. 1F, G), but there was no obvious alteration in the total AMPKα level in the hippocampus after various doses of metformin treatment (F (4, 20) = 0.4209, P = 0.7917; Fig. 1F, G). Additionally, we examined the duration of the antidepressant-like effect induced by metformin. The time course of the behavioral antidepressant-like effects after a single injection of 50 mg/kg metformin according to the FST and TST was measured. After 3 h, 1 day, or 5 days, but not after 7 days, marked reductions in the immobility time in the FST and TST in each of the metformin-injected groups compared to the vehicle-treated group (F (4, 30) = 5.715, P = 0.0015 for FST; F (4, 30) = 10.66, P < 0.001; Fig. 2A, B), indicating that metformin produces fast-acting as well as long-lasting (at least 5 days) antidepressant-like responses.

### Metformin ameliorated chronic restraint stress-induced depressive-like behavior

**Figure 3.**
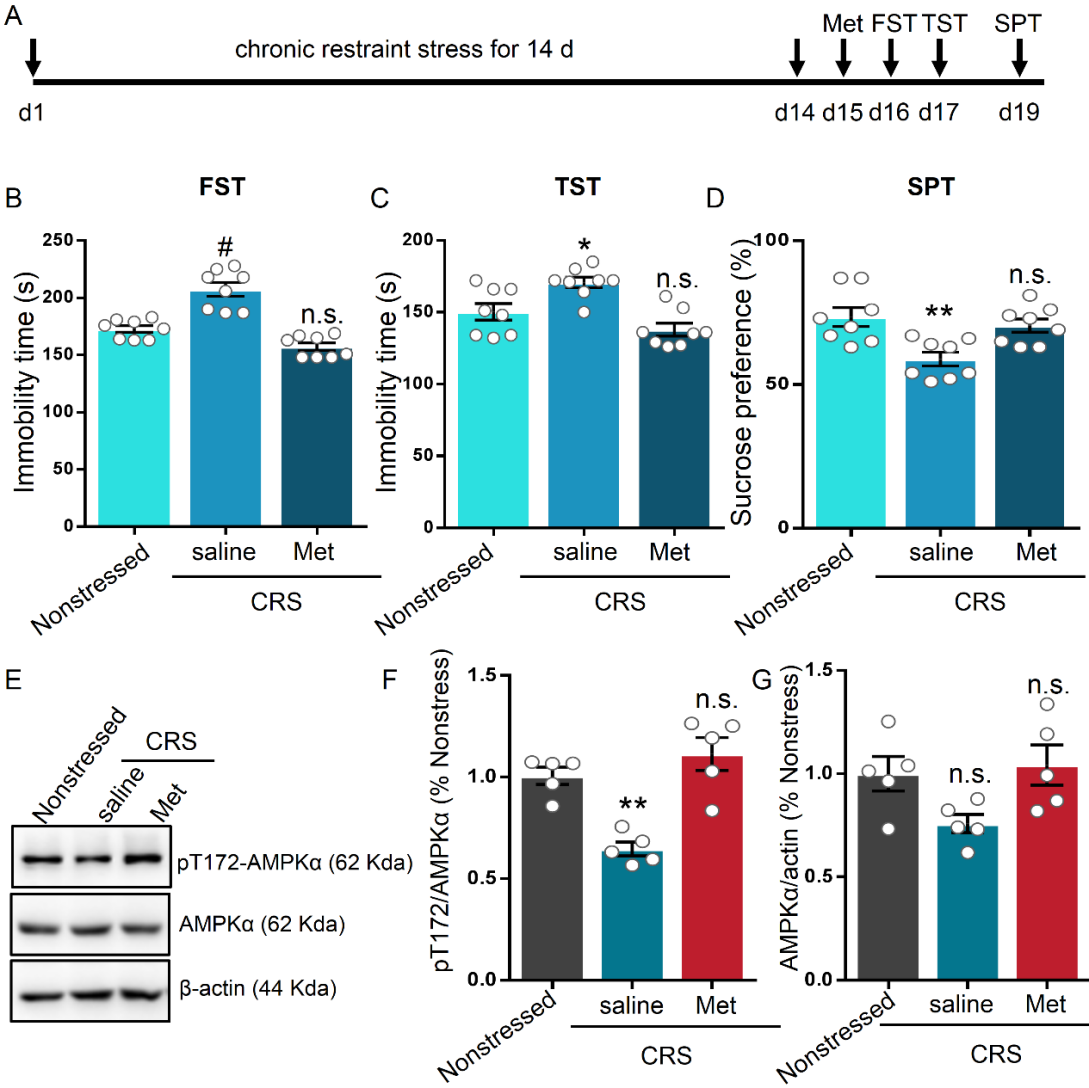
**Metformin produced rapid antidepressant-like effects and diminished the chronic restraint stress (CRS)-induced reduction of p-AMPK in the hippocampus**. **(A)** Timeline of CRS exposure, metformin administration and behavioral test (n = 8 mice per group). **(B-C)** Mean immobility time ± SEM in non-stressed and stressed mice in FST (B) and TST (C). **(D)** Metformin prevented the decrease on the sucrose preference test (SPT) in stressed mice. E. Representative western blot of hippocampal proteins. **(F-G)** Statistical analysis of pT172 (F) and AMPKα (G) levels in the hippocampus, n = 5 mice per group. One-way analysis of variance (ANOVA) with Bonferroni post-hoc analysis. *P < 0.05, **P < 0.01, n.s. represents not significant. Data are presented as mean ± SEM.

Chronic stress, i.e., chronic restraint stress (CRS), triggers pathophysiological modification of experimental rodents, leading to depressive-like behavior [40-42]. Given the establishment of the antidepressant-like effect of metformin in naïve mice, we first determined whether metformin prevent stress-induced depressive-like behavior in mice subjected to chronic restraint stress model. We verified the CRS model by measuring the corticosterone level in non-stressed and stressed mice. The glucocorticoid level in the stressed mice was two-fold higher than that in non-stressed mice, indicating that our CRS model is effective (Supplemental Fig. 1). One day after 14 days CRS, saline and metformin (50mg/kg) was intraperitoneally injected at an equal volume into the stressed mice, respectively (Fig. 3A). Metformin-treated stressed mice exhibited reduced immobility time during the FST compared to that exhibited by saline-treated stress mice (F (2, 21) = 34.36, P < 0.001), but the immobility time of metformin-treated stressed mice was comparable to that of non-stressed mice (Fig. 3B). Consistent with the FST results, metformin-treated stressed mice struggled longer in the TST as compared with saline-treated stressed mice (F (2, 21) = 12.43, P < 0.001; Fig. 3C). In the sucrose preference test (SPT), a measure of anhedonia that is not dependent on locomotor activity, metformin-treated stressed mice favored sucrose over water significantly more than did saline-treated stress mice (F (2, 21) = 8.158, P = 0.0024; Fig. 3D). Considering that metformin treatment regulated the pT172 level, we next explored changes in pT172 and AMPKα levels in the hippocampus after metformin injection in stressed mice without behavioral test. In response to metformin, the pT172 level was comparable to that in non-stressed mice 1 h after metformin injection in stressed mice, although the pT172 level was reduced by nearly 50% in saline-treated stressed mice compared to that in non-stressed and metformin-treated stressed mice (F (2, 12) = 18.84, P < 0.001; Fig. 3E, F). The AMPKα level in the hippocampus was unaltered in either saline- or metformin-treated stressed mice relative to non-stressed mice (F (2, 12) = 3.815, P=0.0522; Fig. 3E, G). These findings suggest that metformin treatment after CRS alleviates stress-induced depressive-like behavior and reduction in the pT172 level in mice.

**Figure 4.**
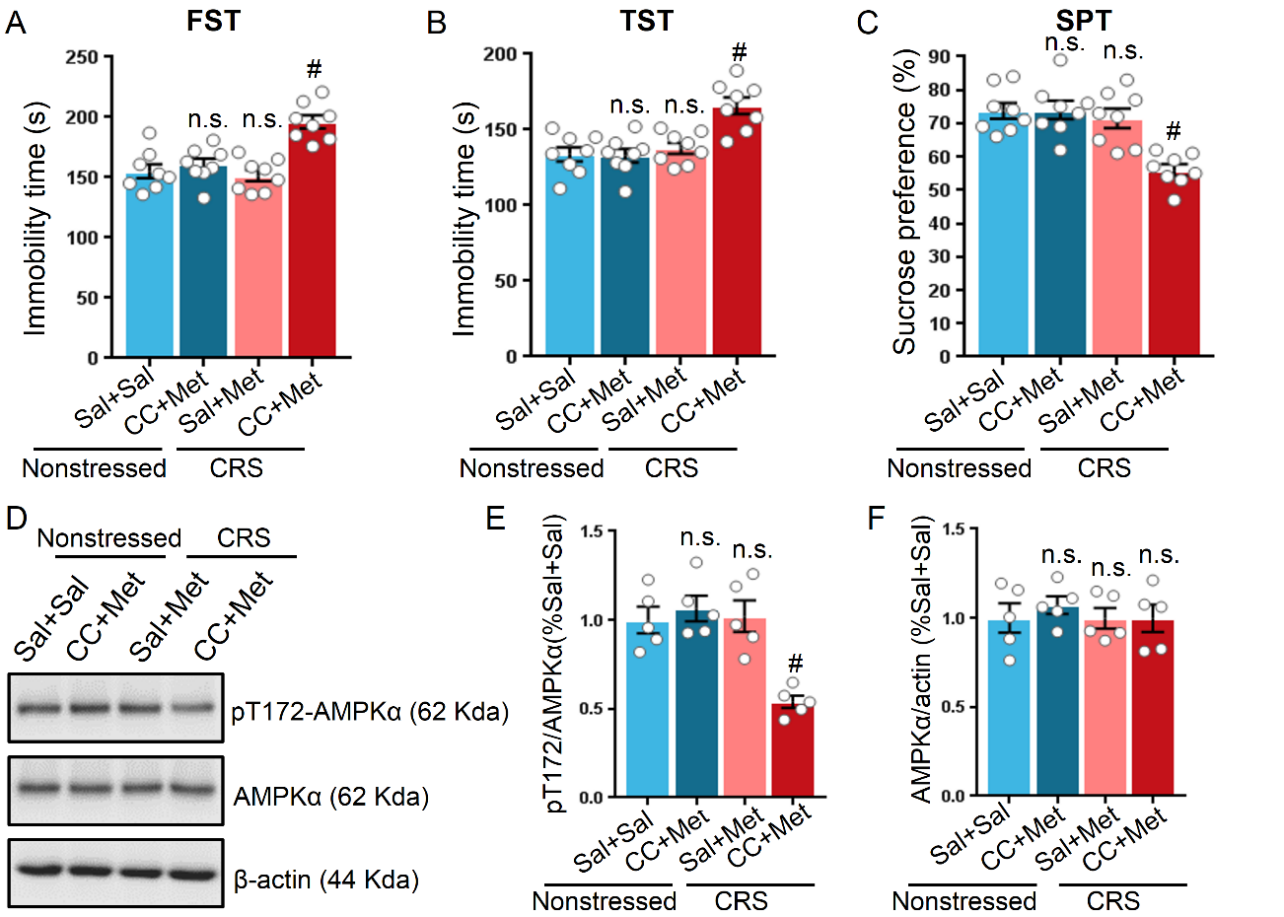
**Inhibition of AMPK by compound C prevented the metformin-induced antidepressant-like effect in stressed mice**. Compound C (CC) attenuated the ability of metformin to reduce the immobility time of mice in FST (A) and TST (B). **(C)** CC abolished the metformin-induced elevation of sucrose preference in SPT as compared with metformin-treated stressed mice (n = 8 mice per group for all behavioral tests). **(D)** Representative western blot of hippocampal proteins in non-stressed or stressed mice treated with metformin or CC plus metformin. Quantification of p-AMPK at T172 (E) and AMPK (F), n = 5 mice per group. One-way ANOVA with Bonferroni post-hoc analysis. ^#^P < 0.001, n.s. represents not significant. Data are presented as mean ± SEM.

### Inhibition of AMPK by compound C prevented the antidepressant effect of metformin

The pT172 level of AMPK coincided with increased struggle time in the FST and TST in stressed mice; this observation prompted us to question whether AMPK activity is required in the antidepressant-like effect induced by metformin. Since compound C has been validated as a drug that inhibits AMPK activity *in vivo* [18], we sought to test the hypothesis that AMPK is indispensable on the antidepressant-like effect of metformin. Application of compound C alone by intraperitoneal injection had a minimal impact on locomotor activity as measured by the OFT, and immobility time during the FST and TST in non-stressed mice (Supplemental Fig. 2). However, administration of compound C 30 min before metformin injection attenuated the antidepressant effect of metformin in non-stressed mice (Fig. 4A-C). Moreover, compound C diminished the effects of metformin in stressed mice, as determined by the elevated immobility time compared to that of non-stressed mice in the FST and TST on the second and third day after drug treatment (F (3, 28) = 14.99, P < 0.001 for FST; F (3, 28) = 11.46, P < 0.001 for TST; Fig. 4A, B). In the SPT, the metformin-injected stressed mice favoring sucrose were hindered by compound C (F (3, 28) = 12.07, P < 0.001; Fig. 4C). In addition to behavior test, we also evaluated the pT172 and AMPK levels in non-stressed and stressed mice receiving with different drug combinations. Notably, the pT172 level in stressed mice treated with compound C plus metformin was reduced to approximately 50% of the pT172 level in non-stressed and stressed mice treated with metformin (F (3, 16) = 12.07, P < 0.001; Fig. 4D, E). The AMPKα levels were indistinguishable among the four groups (F (3, 16) = 0.2921, P = 0.8305; Fig. 4D, F). Taken together, these results support the notion that AMPKα is involved in the antidepressant-like effect induced by metformin.

**Figure 5.**
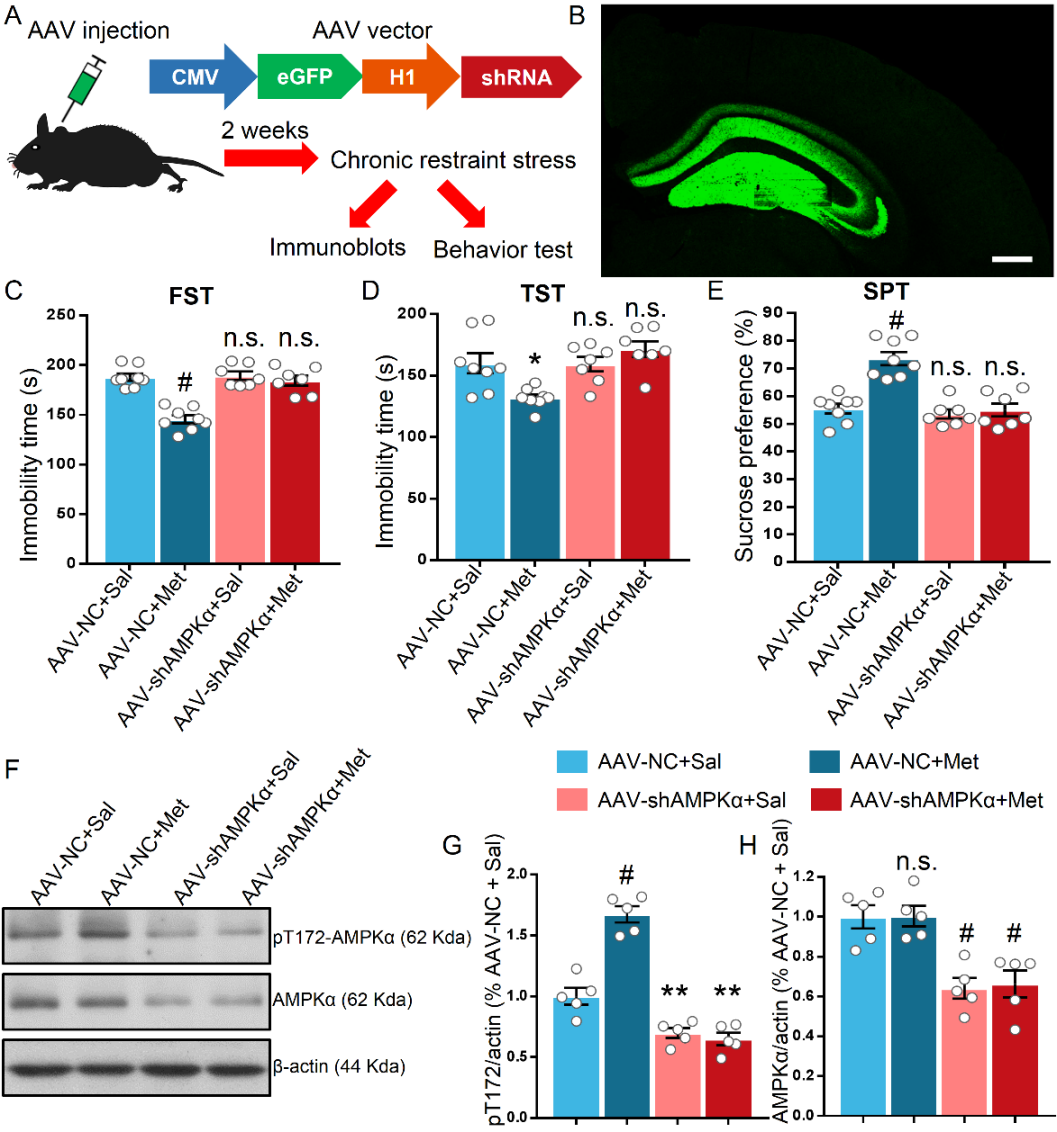
**Adeno-associated virus (AAV)-mediated knock down (KD) of AMPKα in the hippocampus occluded the antidepressant-like effect in stressed mice**. **(A)** Schematic showing the experimental design and targeting strategy for knock down AMPKα. The eGFP (enhanced green fluorescent protein) was exploited to visualize the infection of the virus and an H1 promoter was used to drive the expression of shRNA. **(B)** Representative images of coronal slice with intensive GFP signal, indicating successful AAV infection in the hippocampus, scale bar = 300μm. **(C-D)** KD of AMPKα in the hippocampus hampered the metformin-induced elevation in struggling time in FST (C) and TST (D) of stressed mice, n = 7-8 mice per group. **(E)** KD of AMPKα in the hippocampus occluded the increased sucrose preference in SPT induced by metformin in stressed mice. **(F)** Representative blots of hippocampal proteins in AAV-NC- and AAV-shRNA-injected mice after CRS without behavior test. **(G-H)** Statistical analysis of the pT172 (G) and AMPKα (H) levels in the hippocampus, n = 5 mice per group. The results were analyzed using a two-way ANOVA with Bonferroni post hoc analysis. *P < 0.05, **P < 0.01, n.s. represents not significant. Data are presented as mean ± SEM.

### Knock down of AMPKα in the hippocampus occluded the antidepressant effect of metformin

Given that compound C is not the sole inhibitor for AMPK [43], it is necessary to further elucidate the role of AMPKα in the behavioral impact of metformin. Thus, we performed bilateral stereotactic injection of AAV constituting shRNA against AMPKα into the dorsal hippocampus to demonstrate the requirement of hippocampal AMPKα in the antidepressant-like effect of metformin (Fig. 5A). After 2 weeks of AAV injection, a robust green fluorescent protein (GFP) signal was detected in coronal slice containing the dorsal hippocampus (Fig. 5B). We further verified the efficiency of AMPK inhibition by AAV-mediated knock down. Phosphorylated ACC at serine 79, which is an indicator of the activity of AMPK, was reduced in AAV-shRNA-injected mice compared to that in AAV-NC-injected mice two weeks after AAV injection, while total ACC level was unaltered (Supplemental Fig. 3). Consequently, the ability of metformin in decreasing the immobility time of FST and TST was attenuated by knock down of hippocampal AMPKα (F (1, 26) = 20.9, P < 0.001 for FST; F (1, 26) = 11, P = 0.0027 for TST; Fig. 5C, D). Consistently, knock down of AMPKα in the hippocampus abolished the impact of metformin in stressed mice favoring sucrose in the SPT as compared to AAV-NC-injected mice (F (1, 26) = 16.28, P < 0.001; Fig. 5E). Additionally, we evaluated the pT172 and AMPKα levels in the hippocampus 2 weeks after AAV injection. In AAV-NC-injected mice, metformin treatment led to a significant increase in the pT172 level as compared to that in AAV-NC-injected mice treated with saline (F (1, 16) = 38.57, P < 0.001; Fig. 5F, G). However, there was no overt alteration of the pT172 level of AAV-shRNA-injected mice treated with saline or metformin (Fig. 5F, G). The AMPKα level in AAV-shRNA-injected mice was reduced to approximately 65% as that in AAV-NC-injected mice, indicating successful knock down of AMPKα in the hippocampus (F (1, 16) = 36.49, P < 0.001; Fig. 5F, H). Collectively, the loss of function results suggests that hippocampal AMPKα is critical for the metformin-mediated antidepressant-like effects in the mice behavior.

## DISCUSSION

**Figure 6.**
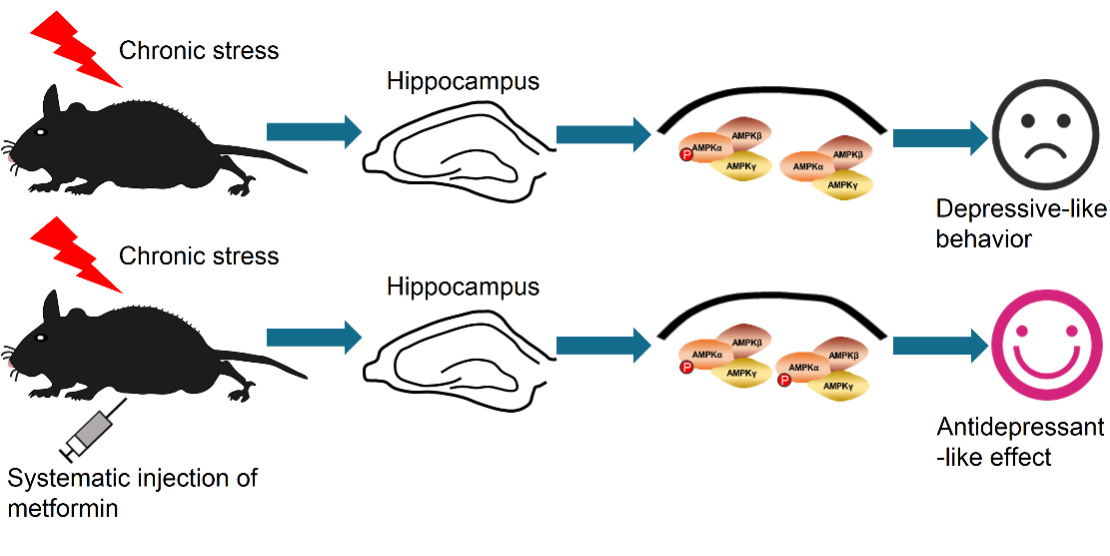
**Summary of our findings. Mice subjected to chronic restraint stress (CRS) that did or did not receive metformin show distinct behavior**. Metformin reversed the reduction of p-AMPK (pT172) level in the hippocampus of mice subjected to CRS, thereby alleviating the depressive-like behavior caused by CRS.

In the current study, we have demonstrated that metformin, a widely used anti-diabetes drug, provides antidepressant-like effects in naïve mice as well as stressed mice. As indicated in the results, different doses of metformin decreased the immobility time of naïve mice in the FST and TST while locomotor activity was unaffected as determined by the OFT. Additionally, the effect of metformin was fast-acting (within hours) and lasted for at least 5 days after a single injection. We further demonstrated that the ability of metformin in decreasing the immobility time in the FST and TST was dependent on AMPK activity. Moreover, inhibition of AMPKα by compound C or knock down of AMPKα in the hippocampus abolished the antidepressant-like effect of metformin, indicating that hippocampal AMPKα is necessary for the effect provided by metformin (see Fig. 6 for a summary).

The neural circuitry responsible for depressive-like behavior most likely involves several brain regions, including the medial prefrontal cortex, hippocampus and multiple components of the limbic system [2, 44-46]. Here, we identified that hippocampal AMPK was critical for the antidepressant-like effect of metformin, notwithstanding other brain regions may also be involved in the effect with rapid onset induced by metformin. Thus, it will be of interest to determine whether additional brain regions participate in the metformin-mediated effect in the subsequent investigations.

It should be noted that there are several stress models, such as chronic unpredictable mild stress or chronic social defeat stress [47-51], that can elicit depressive-like behavior in mice. Here, we exploited chronic restraint stress in the present study. These animal models are extensively and widely utilized for antidepressant drug screens or pinpointing the neural circuitry responsible for depression. However, there has been substantial criticism of these models, and the models are generally faulted for not effectively modeling depression in humans. Nevertheless, accepting the caveats of animal depression models caused by stress and employment of them is a convenient and valuable preliminary step to uncover potential therapeutic targets in the treatment of depression.

The search for pharmacological agents that block various molecular targets with fast-acting effects has led to a number of discoveries [52-55]. Here, we have exploited a different way; we focused on a widely used antidiabetic drug, metformin, rather than searching for novel chemicals or drugs. Metformin has been widely used in humans as a drug for type II diabetes for several decades. There is amounting interest in the notion that recruitment of metformin might provide an alternative therapeutic strategy for the treatment of neurological diseases. Indeed, metformin has been demonstrated to increase the lifespan and delay aging in a number of experimental animals [56, 57], and is reported to provide beneficial impacts on age-related disorders, such as Alzheimer’s disease [15] and ischemic stroke [18], although contradictory reports exist [58]. Intriguingly, a recent study has highlighted the possibility that metformin may alleviate autism-spectrum disorders in a mouse model of fragile X syndrome [39].

It is reported that metformin activates AMPK *in vivo* [30, 59]. AMPK is an evolutionarily conserved serine/threonine kinase sensing metabolic changes, including ATP depletion and metabolic stress [25]. Beyond its role in metabolism regulation, accumulating evidence demonstrates that AMPK plays an important role in neural development and neurological function as well as in diseases [59-63]. Previous studies indicate that stress down-regulates AMPK activity and in turn impairs cognitive function [29, 64, 65]. In the combination of biochemical and behavioral experiments with compound C and AAV-mediated knock down of AMPK *in vivo*, we provided convergent evidence that AMPK was critical in the antidepressant-like effect of metformin in stressed mice. Additionally, we also found that AICAR, an activator of AMPK [66, 67], ameliorates stress-induced depressive-like behavior, showing reduced immobility time on the FST and TST in mice (data not shown). Our and other’s results strongly suggest that AMPK is a viable therapeutic target for treating depression.

The findings of our study permit the development of novel approaches for treatment of stress-induced disorders. The impact of metformin and activation of AMPK by diverse drugs, i.e., AICAR and A-769662, needs to be explored and understood comprehensively in preclinical and clinical experiments when considering metformin or AMPK as a viable therapeutic drug or target in the treatment of stress-induced mood disorders. Nevertheless, these findings bolster the idea that recruitment of metformin might have a positive impact in at least some neurological disorders.

## Supplementary Materials

The Supplemenantry data can be found online at: www.aginganddisease.org/EN/10.14336/AD.2019.0403
