# Supplementary material for: Antidiabetic Drug Metformin Ameliorates Depressive-Like Behavior in Mice with Chronic Restraint Stress via Activation of AMP-Activated Protein Kinase
Source: Aging Dis. 2020 Feb 1;11(1):31–43. doi: 10.14336/AD.2019.0403 (PMC6961762; doi:10.14336/AD.2019.0403)
Supplement: Supplementary file 1 — The Supplemenantry data can be found online at: www.aginganddisease.org/EN/10.14336/AD.2019.0403 [file AD-11-1-31-s.pdf]

SUPPLEMENTARY DATA

Antidiabetic Drug Metformin Ameliorates Depressive-Like Behavior in Mice with Chronic Restraint Stress via Activation of AMP-Activated Protein Kinase

Heng Ai[1,#], Weiqing Fang[2,#], Hanyi Hu[3], Xupang Hu[4], Wen Lu[5*]

[1]Department of Physiology, Hangzhou Medical College, Hangzhou, Zhejiang, China
[2]Department of Pharmacy, Women's Hospital, School of Medicine, Zhejiang University, Zhejiang, China
[3]Department of Ophthalmology, Sir Run Run Shaw Hospital, Zhejiang University, Hangzhou, China
[4]Department of Neurobiology, Key Laboratory of Medical Neurobiology of Ministry of Health of China, Zhejiang University School of Medicine, Zhejiang, China
[5]Department of Biochemistry and Molecular Biology, Hainan Medical University, Haikou, Hainan, China

© 2019. Ai H et al. Published online at http://www.aginganddisease.org/EN/10.14336/AD.2019.0403

SUPPLEMENTARY DATA

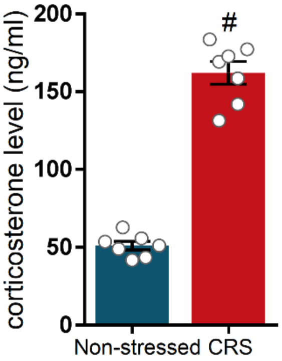

Supplementary Figure 1. Measurement of the corticosterone levels in non-stressed and stressed mice with chronic restraint stress. Data was presented as the mean ± SEM, n = 7 mice per group, unpaired student t test, $^{\#}P < 0.001$.

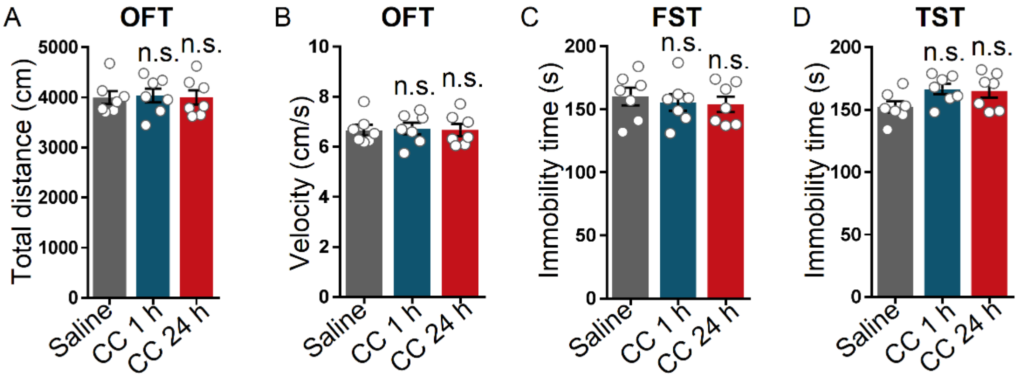

Supplementary Figure 2. Compound C (CC) exerted minimal effects on the mice. A-B. The total distance (A) and the mean velocity (B) measured by the OFT at 1 h or 24 h after injection in the mice treated with saline or compound C (10 mg/kg). C-D. The immobility time in the FST (C) and TST (D) in the mice treated with saline or compound C. One-way analysis of variance (ANOVA) with Bonferroni post hoc analysis, n = 7 mice per group, n.s. represents not significant.

© 2019. Ai H et al. Published online at http://www.aginganddisease.org/EN/10.14336/AD.2019.0403

SUPPLEMENTARY DATA

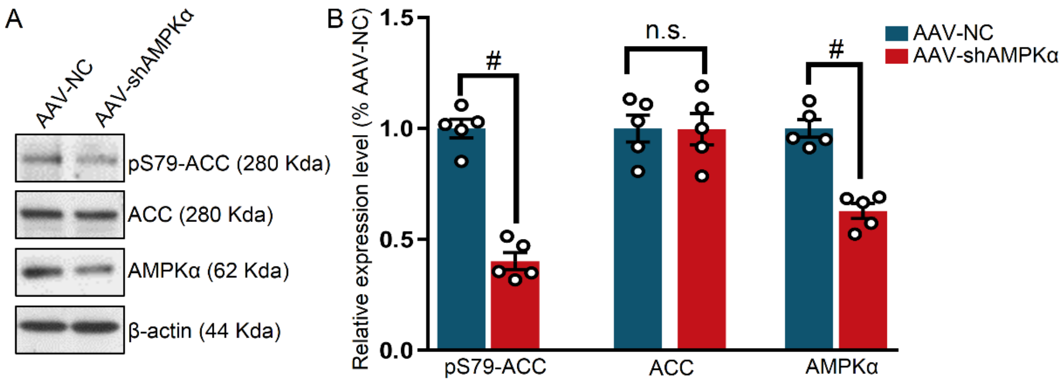

Supplementary Figure 3. Inhibition of AMPK activity by AAV-mediated knock down in the hippocampus. A. Representative blots of hippocampal proteins in AAV-NC- and AAV-shRNA-injected mice. B. Statistical analysis of the pS79-ACC, ACC and AMPKα levels in the hippocampus, n = 5 mice per group. Unpaired student t test, $^{\#}P < 0.001$, n.s. represents not significant.

© 2019. Ai H et al. Published online at http://www.aginganddisease.org/EN/10.14336/AD.2019.0403
